# Linking dynamical complexities from activation signals to transcription responses

**DOI:** 10.1098/rsos.190286

**Published:** 2019-03-27

**Authors:** Genghong Lin, Feng Jiao, Qiwen Sun, Moxun Tang, Jianshe Yu, Zhan Zhou

**Affiliations:** 1 Center for Applied Mathematics, Guangzhou University, Guangzhou, 510006, People’s Republic of China; 2 Department of Mathematics, Michigan State University, East Lansing, MI 48824, USA

**Keywords:** stochastic gene transcription, promoter states, mean transcription level, oscillation and noise filtration, dynamical complexity

## Abstract

The transcription of inducible genes involves signalling pathways that induce DNA binding of the downstream transcription factors to form functional promoter states. How the transcription dynamics is linked to the temporal variations of activation signals is far from being fully understood. In this work, we develop a mathematical model with multiple promoter states to address this question. Each promoter state has its own activation and inactivation rates and is selected randomly with a probability that may change in time. Under the activation of constant signals, our analysis shows that if only the activation rates differ among the promoter states, then the mean transcription level *m*(*t*) displays only a monotone or monophasic growth pattern. In a sharp contrast, if the inactivation rates change with the promoter states, then *m*(*t*) may display multiphasic growth patterns. Upon the activation of signals that oscillate periodically, *m*(*t*) also oscillates later, almost periodically at the same frequency, but the magnitude decreases with frequency and is almost completely attenuated at high frequencies. This gives a surprising indication that multiple promoter states could filter out the signal oscillation and the noise in the random promoter state selection, as observed in the transcription of a gene activated by p53 in breast carcinoma cells. Our approach may help develop a theoretical framework to integrate coherently the genetic circuit with the promoter states to elucidate the linkage from the activation signal to the temporal profile of transcription outputs.

## 1. Introduction

In living organisms, cells are constantly exposed to environmental or intracellular challenges, such as pathogen invasions, nutritional stresses, developmental cues and transitions in growth phases. In response to these challenges, cells show a remarkable regulatory plasticity to adapt themselves for normal cell functions and physiological homeostasis [1,2]. Gene transcription regulation is a central process in this adaption by activating or repressing the production of messenger RNA (mRNA) molecules of inducible genes. It involves activation of multiple signal transduction pathways, remodelling of chromatin structures and recruitment of transcription factors (TFs) to cognate binding sites in the gene promoter domains [1,3]. Extensive studies in past years have largely contributed to the identification of regulatory factors and biochemical steps in transcriptional regulation [1–4]. However, how the temporal profiles of mRNA level are shaped by the rewire of transcriptional programmes remains to be systematically explored, as it is largely unknown how the nonlinear behaviour of transcription dynamics is linked to the temporal variations of activation signals. The distinct profiles of transcriptional dynamics are strongly tied with the biological functions: The proteins maintaining circadian rhythm or regulating cell cycle progression are often encoded by the genes whose transcription levels oscillate; the proteins involved in immunity are often encoded by the genes whose transcription levels rise fast upon pathogen invasions followed by a slow attenuation; and the proteins involved in the rearrangement of the extracellular matrix are encoded by the genes whose transcription levels rise steadily [3,5–7].

**Figure 1.**
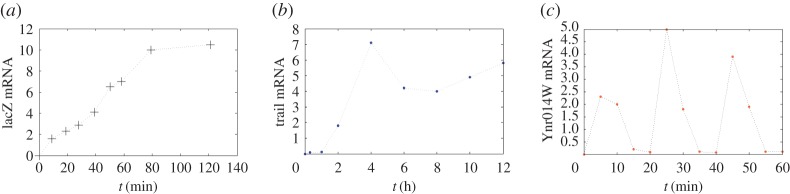
Temporal profiles in the growth of transcript counts. Upon environmental cues, gene transcription levels may follow a (*a*) monotonic [20]; (*b*) biphasic or multiphasic [18] or (*c*) oscillatory growth [4].

The two-state model has been a primary mathematical tool to quantify transcription kinetics in cells, from bacteria [8,9] and yeast [10,11] to mammalian cells [8,12]. In the model, as the following diagram shows,

1.1 $$\text{gene OFF}\overset{\lambda }{\underset{\gamma }{\;⇌}}\text{gene ON}\overset{\nu }{⟶}\text{mRNA}\overset{\delta }{⟶}\emptyset ,$$

the gene is postulated to switch randomly between active (ON) and inactive (OFF) states, with activation rate λ > 0 and inactivation rate *γ* > 0. Transcripts are produced with rate *ν* > 0 when the gene is active and are degraded in rate *δ* > 0 [8,9]. This model has been widely used to fit the experimental data and yields insight into the stochastic gene transcription [8,10,12]. However, the two-state model predicts only simple dynamical behaviour for the growth of transcription level (figure 1*a*). It was shown in [13,14] that if the transcription of a gene follows the two-state model, then the average of mRNA copy numbers will often grow monotonically. In the transcription of genes that respond to environmental stresses such as heat, salinity or osmotic pressure, the growth of transcription level may exhibit more complex dynamical behaviours [3,5]. From yeast [4,15] to mammalian cells [16–18], it has been observed that transcription levels may develop two peaks (figure 1*b*), called biphasic growth in Hager *et al.* [17,19], or even oscillate [4] (figure 1*c*). For those genes, application of the two-state model is inadequate.

The transcription of inducible genes involves the activation of signal transduction pathways that induce the binding of downstream TFs at the cognate DNA binding sites in the gene promoter or enhancer domains. The cooperative or competitive bindings of TFs at these sites result in multiple TF/DNA binding configurations or promoter states [21,22]. In this work, we extend the classical two-state model to a model with multiple promoter states to study the nonlinear transcription dynamics. Each promoter is characterized by its own activation and inactivation rates. The temporal dependence of gene transcription on activation signals is attributed to the time dependence of random promoter state selection. At time *t* > 0, the transcription state in a single cell is quantified by the joint probabilities that record the copy number of the transcripts and the usage of each promoter state. The time evolutions of these joint probabilities satisfy a system of master equations, from which we derive the differential equation of the mean transcription level *m*(*t*).

Our mathematical analysis yields two distinct scenarios for *m*(*t*) when the transcription is activated by constant signals that the promoter selection probabilities are time independent: If only the activation rates differ among the promoter states, then *m*(*t*) displays simple dynamics as in the two-state model. In a sharp contrast, if the inactivation rates change with promoter states, then *m*(*t*) may display multiphasic growth patterns, indicating a more prominent role of gene inactivation than activation in shaping transcription profiles. When the transcription is activated by signals that oscillate periodically in time, *m*(*t*) oscillates almost periodically at the same frequency after a short time period of rapid growth, but its oscillation magnitude decreases with frequency and is almost completely attenuated at high frequencies. This gives a surprising indication that an orchestrated interplay between multiple promoter states and periodic signals may produce simple transcription dynamics such as a monotone growth for all *t* > 0, as observed in the transcription of a gene activated by p53 in MCF-7 breast carcinoma cells [23]. It indicates further that multiple promoter states are capable of filtering out both the oscillation in activation signals and the noise in the random switching between the promoter states.

## 2. The model and equations

### 2.1. The model

In the classical two-state model depicted in (1.1), if a gene of our interest is inactive in all cells at a specified initial time *t* = 0 and the residual mRNAs are not counted, then the mean transcription level for *t* ≥ 0 is given by Peccoud and Ycart [24]

2.1 $$m(t)=\frac{\lambda }{\left(\lambda +\gamma \right)}\frac{\nu }{\delta }+\frac{\lambda \nu }{\left(\lambda +\gamma )(\lambda +\gamma -\delta \right)}\;e^{-(\lambda +\gamma )t}+\frac{\lambda \nu }{\delta (\delta -\lambda -\gamma )}\;e^{-\delta t}.$$

It increases from *m*(0) = 0 to approach the steady state λ/(λ+γ)⋅(ν/δ) as *t* → ∞, where λ/(λ + *γ*) is the stationary activation rate, and *ν*/*δ* is the effective synthesis rate [13,14]. When the gene is active in some cells, *m*(*t*) can be expressed similarly as in (2.1) and may either increase for all *t* > 0 or peak uniquely before decaying to the steady state. The simple dynamics exhibited by the two-state model has been widely observed in the genes that are constitutively transcribed or activated by stable environmental signals [8,10,12].

**Figure 2.**
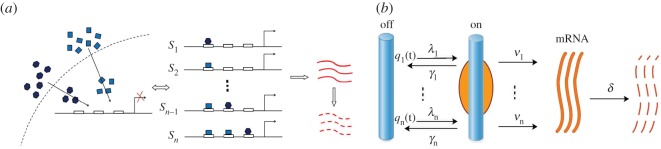
A schematic view of gene transcription activated by multiple promoter states. (*a*) One or several signal transduction pathways converge to the promoter of a target gene. The bindings of their downstream TFs at various DNA binding sites result in *n* promoter states *S*_1_, *S*_2_, … , *S*_*n*_. (*b*) When the gene is turned off at time *t*, the promoter state *S*_*i*_ has a probability *q*_*i*_(*t*) to activate the transcription with constant kinetic rates λ_*i*_, *γ*_*i*_, *ν*_*i*_ and *δ*.

Upon environmental stimuli, the transcription of inducible genes may display complex dynamics such as biphasic, multiphasic or oscillatory growth of transcription levels as seen in figure 1*b*,*c* [3–6,19]. Usually, the activation of inducible genes involves two or more signal transduction pathways. For instance, the yeast glucose-regulated SUC2 gene is activated by AMPK/Snf1 and cAPK signalling pathways [15], and the mouse macrophage genes stimulated by lipopolysaccharide are activated by p38 MAPK and JNK pathways [16]. In these cases, a direct application of the two-state model is apparently inadequate. To model these transcriptional complexities, we note that the activation by signal transduction pathways is ultimately mediated through the binding of downstream transcription factors (TFs) at the cognate DNA binding sites in the gene promoter or enhancer domains (figure 2*a*). For each target gene, there are often multiple DNA binding sites recognizable by distinct TFs. The bindings of different TFs or multiple copies of the same TF at these sites may result in a large number of TF/DNA binding configurations [21,22]. In the lysogeny maintenance promoter of bacteriophage lambda *P*_*RM*_, hundreds of TF/DNA configurations by lambda repressor binding were observed [21,25]. By modulating transcription frequencies, durations and amplitudes, the combinatory TF/DNA interactions in these configurations play essential roles in determining the transcriptional output [26,27].

We are motivated by these observations to study the nonlinear dynamics of stochastic gene transcription regulated by multiple TF/DNA configurations [28–30]. Although the number of TF/DNA configurations for one gene could be large by combinatorics, some configurations may have redundant functions to induce similar transcription activities, and many others may be highly unstable [21,22,25]. By ignoring the unstable configurations, and then placing the configurations with similar functions into one group, called a *promoter state* in [22], the total number could be greatly reduced. In [22], the hundreds of configurations in the lysogeny maintenance promoter of bacteriophage lambda *P*_*RM*_ were reduced to three promoter states. In our model, as depicted in figure 2, we assume

(H1) There are *n* promoter states, labelling as *S*_1_, *S*_2_, … , *S*_*n*_, with distinct functions to activate or repress the transcription of a target gene. When the gene is turned off at time *t*, the promoter state *S*_*i*_ has a probability *q*_*i*_(*t*) to activate the transcription.
(H2) If the transcription is regulated by the promoter state *S*_*i*_, then the transcription follows the classical two-state model with constant activation rate λ_*i*_, inactivation rate *γ*_*i*_ and production rate *ν*_*i*_. The transcripts are degraded at a uniform constant rate *δ*.

We call a promoter state *S*_*i*_, along with the triplet (λ_*i*_, *γ*_*i*_, *ν*_*i*_) of parameters, a *transcription mode*. If the transcription of the target gene is regulated by a single mode (*n* = 1), then our model is reduced to the classical two-state model. In this case, the triplet (λ_1_, *γ*_1_, *ν*_1_) and *δ* have been estimated by using the MS2-GFP method to measure the real-time transcription kinetics [20], or the single-molecule fluorescence *in situ* hybridization (smFISH) method to generate the histogram of mRNA copy-numbers at steady state [8].

In general, if the transcription is regulated by multiple modes (*n* > 1), then we need to estimate the relative probability *q*_*i*_(*t*) for each promoter state satisfying

2.2 $$q_{i}(t)\in [0,1],\;i=1,2,\ldots ,n\;\text{and}\;\sum_{i=1}^{n}q_{i}(t)=1.$$

Determining these probabilities is rather challenging, which involves the accessibility of the promoter, the availability of TFs, the cooperation or competition of TFs and the stability of promoter states. In [21,22,25], they are approximated by integrating the concentrations of TFs into an equilibrium thermodynamic model. In our analysis below, we will adapt the equilibrium thermodynamic model [21,22,25] to estimate *q*_*i*_(*t*): in the simplest case when all promoter states are formed by the same TF,

2.3 $$q_{i}(t)=\frac{a_{i}{\left[\text{TF}(t)\right]}^{n_{i}}}{\sum_{\;j=1}^{n}a_{j}{\left[\text{TF}(t)\right]}^{n_{j}}},$$

where [TF] is the concentration of free TFs in the cell, *n*_*i*_ is the number of TFs bound to DNA in the state *S*_*i*_, and the constant *a*_*i*_ is the Boltzmann weight of *S*_*i*_. To determine (λ_*i*_, *γ*_*i*_, *ν*_*i*_), genetic control strategies have been used by knocking down or blocking some DNA binding sites, manipulating the concentrations or silencing the functions of some TFs, so that only one of *S*_1_, *S*_2_, … , *S*_*n*_ could be formed in the promoter [22,26].

### 2.2. The differential equation of the mean transcription level

Let *t* ≥ 0. We define a random process *M*(*t*) to count the copy number of the mRNA molecules of a target gene in a single cell and define another random process *X*(*t*) to specify the transcription state with *X*(*t*) = *O*_*i*_ if the gene is OFF, but its transcription is activating by the promoter state *S*_*i*_, and *X*(*t*) = *E*_*i*_ if the gene is ON and the transcription is turned on by *S*_*i*_. The transcription can be characterized by the joint probabilities

2.4 $$P_{0,i}(m,t)=\text{Prob}\{M(t)=m,\;X(t)=O_{i}\},\;i=1,2,\ldots ,n,\;m=0,1,2,\ldots$$

and

2.5 $$P_{1,i}(m,t)=\text{Prob}\{M(t)=m,X(t)=E_{i}\},\;i=1,2,\ldots ,n,\;m=0,1,2,\ldots .$$

The master equations that determine the time evolutions of these probabilities are derived in the electronic supplementary material. Without loss of generality, we assume that the gene is inactive in all cells at an initial time *t* = 0, and the residual mRNAs at *t* = 0 are neglected. It gives the initial condition

2.6 $$P_{0,i}(0,0)=q_{i}(0),\;P_{0,\;i}(m,0)=0\;\text{for}\;m>0\;\text{and}\;P_{1,i}(m,0)=0\;\text{for}\;m\geq 0.$$

The mean transcription level *m*(*t*) is given by

2.7 $$m(t)=E(M(t))=\sum_{i=1}^{n}\sum_{k=0}^{\infty }k[P_{0,i}(k,t)+P_{1,i}(k,t)].$$

By introducing the probabilities

2.8 $$P_{0,i}(t)=\sum_{m=0}^{\infty }P_{0,i}(m,t)\;\text{and}\;P_{1,i}(t)=\sum_{m=0}^{\infty }P_{1,i}(m,t),$$

that the transcription is regulated or activated by the promoter state *S*_*i*_, and using the initial condition (2.6), we obtain the following self-contained system of differential equations:

2.9 $$\frac{\text{d}P_{0,i}(t)}{\text{d}t}=-\lambda_{i}P_{0,i}(t)+q_{i}(t)\sum_{\;j=1}^{n}\gamma_{j}P_{1,j}(t),\;P_{0,i}(0)=q_{i}(0),$$

2.10 $$\frac{\text{d}P_{1,i}(t)}{\text{d}t}=\lambda_{i}P_{0,i}(t)-\gamma_{i}P_{1,i}(t),\;P_{1,i}(0)=0$$

2.11 $$\;\text{and}\;\frac{\text{d}m(t)}{\text{d}t}=-\delta m(t)+\sum_{i=1}^{n}\nu_{i}P_{1,i}(t),\;m(0)=0.$$

The derivations of (2.9)–(2.11) are presented in the electronic supplementary material.

## 3. The transcription dynamics with constant promotor state probabilities

Before investigating how the nonlinear dynamical complexity of environmental signals is carried over to the transcription level, we consider an extreme case that the environmental conditions are stable and the promoter state probabilities *q*_*i*_(*t*) remain constants. This case could occur when the promoters are readily accessible and the regulating TFs are plentiful in all cells. We let *q*_*i*_(*t*) = *q*_*i*_, *i* = 1, 2, … , *n*, and consider separately two cases: The transcriptional stability and the mRNA production rate are independent of the promoter state, or the production rate remains independent of the promoter state but the stability changes with promoter states.

### 3.1. Case 1: *γ*_*i*_ = *γ* and *ν*_*i*_ = *ν*

By labelling the transcription activation rates appropriately, we may assume

3.1 $$0<\lambda_{1}<\lambda_{2}<\cdots <\lambda_{n-1}<\lambda_{n},\;q_{i}(t)=q_{i},\;\gamma_{i}=\gamma ,\;\nu_{i}=\nu ,\;i=1,2,\ldots ,n.$$

Under this condition, we show that *multiple promoter states produce essentially the same transcription dynamics as the classical two-state model does*. The mean transcription level either grows monotonically for all *t* > 0 to approach the steady state as *t* → ∞, or grows initially until reaching a unique peak and then decays to the steady state.

A rigorous statement of the main result is inevitably technical. It involves the polynomial

3.2 $$h(x)=\sum_{i=1}^{n}q_{i}\lambda_{i}\prod_{\;j\neq i}(\lambda_{j}-x).$$

The ordering of λ_*i*_ in (3.1) gives *h*(λ_*i*_) > 0 if *i* is odd, and *h*(λ_*i*_) < 0 if *i* is even, *i* = 1, 2, … , *n*. It follows that all the *n* − 1 roots of *h*(*x*) = 0 are real, distinct and lie within (λ_1_, λ_*n*_). The probability that the gene is active at a time *t* is given by

3.3 $$P_{E}(t)=\sum_{i=1}^{n}P_{1,i}(t),$$

where *P*_1, *i*_(*t*) is defined in (2.8). It can be solved from the homogeneous linear system

3.4 $$\begin{array}{rl} \frac{\text{d}P_{0,k}(t)}{\text{d}t} & =-\lambda_{k}P_{0,k}(t)+q_{k}\gamma P_{E}(t)\\ \;\text{and}\;\frac{\text{d}P_{E}(t)}{\text{d}t} & =\sum_{i=1}^{n}\lambda_{i}P_{0,i}(t)-\gamma P_{E}(t), \end{array}\}$$

with the initial conditions *P*_*E*_(0) = 0, *P*_0,*k*_(0) = *q*_*k*_ for *k* = 1, 2, … , *n*.

**Theorem 3.1.**

*Let condition* *(3.1)* *hold. If the smallest zero* *x*_1_ > 0 *of* *h*(*x*) *and the eigenvalue* −*α*_1_ *of the coefficient matrix* *B* *of* *(3.4)* *satisfy* *x*_1_ ≥ min{*δ*, *α*_1_}, *then* *m*′(*t*) > 0 *for all* *t* > 0. *If* *x*_1_ < min{*δ*, *α*_1_}, *then there is a finite* *t*_*m*_ > 0 *such that* *m*(*t*) *increases for* *t* ∈ (0, *t*_*m*_), *peaks at* *t*_*m*_, *and decreases for* *t* > *t*_*m*_.

A mathematical proof of theorem 3.1 is given in the electronic supplementary material. It gives a complete classification for the dynamics of the mean transcription level *m*(*t*) by the relation between *x*_1_ and min{*δ*, *α*_1_}. It is, however, not directly applicable since *x*_1_ and *α*_1_ cannot be expressed as simple functions of the kinetic parameters. In the following result, we provide a weaker, but more easily applicable, criterion to determine the dynamics behaviour of *m*(*t*).

**Corollary 3.2.**

*Let condition* *(3.1)* *hold. If* λ_1_ ≥ min{*δ*, *γ*}, *then* *m*′(*t*) > 0 *for all* *t* > 0. *If* λ_2_ ≤ min{*δ*, *γ*}, *then* *m*(*t*) *peaks uniquely at a finite time*.

### 3.2. Case 2: two promoter states with *γ*_1_ ≠ *γ*_2_ and *ν*_1_ = *ν*_2_ = *ν*

When the transcriptional stability and the mRNA production rate are independent of the promoter state, it has been shown in theorem 3.1 that the mean transcription level either grows monotonically for all *t* > 0 or peaks uniquely at a finite time. In what follows, we will show that when the inactivation rates change with the promoter states, the transcription dynamics can be much more complicated comparing to the simple dynamics revealed in theorem 3.1. Since it is technically formidable to find the analytical form of *m*(*t*) for arbitrarily many promoter states, we focus on the simplest case with two promoter states only. Assume

3.5 $$n=2,\;q_{1}(t)=q_{1},\;q_{2}(t)=q_{2},\;\gamma_{1}\neq \gamma_{2},\;\nu_{1}=\nu_{2}=\nu .$$

In this case, we find that multiple promoter states could generate complex transcription dynamics. Even with two promote states, *m*(*t*) may develop a biphasic growth pattern with two peaks not seen in the classical two-state model.

We rewrite (2.9) and (2.10) as

3.6 $$\begin{array}{rl} \frac{\text{d}P_{0,1}(t)}{\text{d}t} & =-\lambda_{1}P_{0,1}(t)+q_{1}\gamma_{1}P_{1,1}(t)+q_{1}\gamma_{2}P_{1,2}(t),\\ \frac{\text{d}P_{0,2}(t)}{\text{d}t} & =-\lambda_{2}P_{0,2}(t)+q_{2}\gamma_{1}P_{1,1}(t)+q_{2}\gamma_{2}P_{1,2}(t),\\ \frac{\text{d}P_{1,1}(t)}{\text{d}t} & =\lambda_{1}P_{0,1}(t)-\gamma_{1}P_{1,1}(t)\\ \;\text{and}\;\frac{\text{d}P_{1,2}(t)}{\text{d}t} & =\lambda_{2}P_{0,2}(t)-\gamma_{2}P_{1,2}(t) \end{array}\}$$

with the initial conditions *P*_0,1_(0) = *q*_1_, *P*_0,2_(0) = *q*_2_ and *P*_1,1_(0) = *P*_1,2_(0) = 0, and denote by *C* the matrix of constant coefficients for (3.6). As *n* = 2, we rewrite (2.11) as

3.7 $$\frac{\text{d}m(t)}{\text{d}t}=-\delta m(t)+\nu P_{1,1}(t)+\nu P_{1,2}(t),\;m(0)=0.$$

It can be verified that zero is a simple eigenvalue of *C*. Let −*α*_1_, −*α*_2_ and −*α*_3_ denote the three non-zero eigenvalues of *C*. The delicate relation of these eigenvalues is classified in the electronic supplementary material, including in particular, an explicit condition ensuring

3.8 $$0<\alpha_{1}<\alpha_{2}<\alpha_{3}.$$

Let *x*_1_ and *x*_2_ denote the two roots of

3.9 $$H(x)=\nu q_{1}\lambda_{1}(\lambda_{2}-x)(\gamma_{2}-x)+\nu q_{2}\lambda_{2}(\lambda_{1}-x)(\gamma_{1}-x)=0.$$

It is possible that both *x*_1_ and *x*_2_ are complex numbers.

**Theorem 3.3.**

*Let conditions* *(3.5)* *and* *(3.8)* *hold. Assume that* *δ* ≠ *α*_*i*_ *for* *i* = 1, 2, 3. *If* *x*_1_ *and* *x*_2_ *are complex valued, then either* *m*(*t*) *increases monotonically for all* *t* > 0, *or* *m*(*t*) *develops two peaks in a biphasic growth. If* *x*_1_ *and* *x*_2_ *are real valued with* *x*_1_ < *x*_2_ < *α*_3_, *then we have*:

(1) *If either* *x*_1_ < *α*_1_ < *x*_2_ *and* *x*_1_ < *δ*, *or* *x*_1_ < *δ* < *x*_2_ < *α*_1_, *then* *m*(*t*) *increases initially until reaching a peak and then goes down*.
(2) *If one of the following occurs*: *(i)* *δ* < *x*_1_ < *α*_1_ < *x*_2_, *(ii)* *α*_1_ < *x*_1_ < *δ* < *x*_2_ < *α*_2_, *(iii)* *α*_2_ < *δ* < *x*_1_, *and* *(iv)* *α*_1_ < *x*_1_ < *α*_2_ < *x*_2_, *then* *m*′(*t*) > 0 *for all* *t* > 0.
(3) *If one of the following occurs*: *(i)* *x*_2_ < min{*α*_1_, *δ*}, *(ii)* max{*α*_2_, *δ*} < *x*_1_, *(iii)* max{*α*_1_, *δ*} < *x*_1_ < *x*_2_ < *α*_2_, *(iv)* *δ* < *x*_1_ < *x*_2_ < *α*_1_, (*v*) *α*_2_ < *x*_1_ < *x*_2_ < *δ*, (*vi*) *α*_1_ < *x*_1_ < *x*_2_ < min{*α*_2_, *δ*}, *then either* *m*(*t*) *increases monotonically for all* *t* > 0, *or* *m*(*t*) *develops two peaks in a biphasic growth*.

A mathematical proof of theorem 3.3 is given in the electronic supplementary material. We use an example to demonstrate the three different transcription dynamical behaviours described in theorem 3.3. It was found in [31] that the transcription of lacZ gene in *Escherichia coli* is regulated by a basal promoter state *S*_1_ and an active promoter state *S*_2_, with the promoter probabilities estimated at *q*_1_ = 0.4 and *q*_2_ = 0.6. As suggested by the measurements in [9], the production rates of mRNAs are approximately *ν*_1_ = *ν*_2_ = 15 min^−1^, and the degradation rate is *δ* = 0.5 min^−1^. If we take λ_1_ = 0.027 min^−1^ and *γ*_1_ = 0.17 min^−1^ suggested by [20], and λ_2_ = 2.16 min^−1^ and *γ*_2_ = 3.68 min^−1^ suggested by [22], then

$$\alpha_{1}\approx 0.205,\;\alpha_{2}\approx 0.600,\;\alpha_{3}\approx 5.232,\;x_{1}\approx 0.122+0.235\;i,\;x_{2}\approx 0.122-0.235\;i.$$

**Figure 3.**
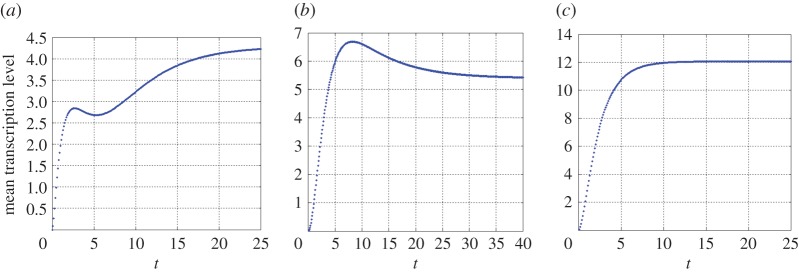
Multiple growth patterns of transcription dynamics activated by two promoter states. The three curves are generated by (3.6) and (3.7) with *q*_1_ = 0.4, *q*_2_ = 0.6, *ν*_1_ = *ν*_2_ = 15 min^−1^ and *δ* = 0.5 min^−1^. (*a*) λ_1_ = 0.027, *γ*_1_ = 0.17, λ_2_ = 2.16 and *γ*_2_ = 3.68. (*b*) λ_1_ = 0.027, *γ*_1_ = 0.17, λ_2_ = 0.3, and *γ*_2_ = 0.45. (*c*) λ_1_ = 0.7, *γ*_1_ = 7.5, λ_2_ = 1.1 and *γ*_2_ = 0.86. All units in (*a*–*c*) are min^−1^.

As *x*_1_ and *x*_2_ are complex valued, theorem 3.3 indicates that *m*(*t*) may either increase for all *t* > 0 or develop two peaks. Our numerical simulation further reveals that the second case occurs and *m*(*t*) develops two peaks with a biphasic growth; see figure 3*a*. If we take λ_1_ = 0.027, *γ*_1_ = 0.17, λ_2_ = 0.3 and *γ*_2_ = 0.45, again suggested by [9,20], then *α*_1_ ≈ 0.124, *α*_2_ ≈ 0.153, *α*_3_ ≈ 0.670, *x*_1_ ≈ 0.082, and *x*_2_ ≈ 0.147. It follows that

$$x_{1}<\alpha_{1}<x_{2}<\alpha_{3},\;x_{1}<\delta ,$$

and the first case in (1) of theorem 3.3 occurs. Both our numerical simulation and theorem 3.3(1) show that *m*(*t*) increases initially until reaching a peak and then goes down; see figure 3*b*. If we take λ_1_ = 0.7, *γ*_1_ = 7.5, λ_2_ = 1.1, and *γ*_2_ = 0.86 suggested by [32], then *α*_1_ ≈ 0.729, *α*_2_ ≈ 1.633, *α*_3_ ≈ 7.799, *x*_1_ ≈ 0.704, and *x*_2_ ≈ 5.637. It follows that

$$\delta <x_{1}<\alpha_{1}<x_{2}<\alpha_{3},$$

and the first case in (2) of theorem 3.3 occurs. Both our numerical simulation and theorem 3.3(2) show that *m*(*t*) grows monotonically for all *t* > 0; see figure 3*c*.

## 4. The transcription activated by time-varying signals

Our theorem 3.3 shows that even though a gene is activated by stable signals with constant kinetic rates, the presence of two pathways with different inactivation rates may generate a bumpy transcription response. What could we expect if a gene is activated by time-varying signals in the presence of multiple activation pathways? Naturally, one may envisage that the temporal profiles of the average transcription level would mirror the complexity of the activation signals. Surprisingly, our numerical examples below show that multi-pathways may transform a periodic activation signal into a monotone transcription output. Taken together, our analysis shows that *the cross-talking of multiple signal transduction pathways is able to generate complex transcription dynamics from constant signals or produce simple transcription dynamics from complex signals*. The cross-talking of signalling pathways has a potential of creating complexity from simple sources, or vice versa, producing simplicity from complex sources. Our model provides a theoretical framework to help understand heuristically the delicate linkage between the temporal variations of activation signals and the nonlinear transcription responses.

### 4.1. Promoter probabilities versus activation signals

A primary step in the use of our model is to determine the promoter state probabilities in terms of the strength of activation signals. In the following, we show through an example of how the probabilities can be obtained. Sepúlveda *et al.* [22] monitored the transcription of lacZ gene in individual *Escherichia coli* cells by controlling the lysogeny maintenance promoter of bacteriophage lambda *P*_*RM*_. They grouped the lambda repressor binding configurations in three promoter states: the basal promoter state *S*_1_ with a probability *q*_1_(*t*), the activated promoter state *S*_2_ with a probability *q*_2_(*t*), and the repressed promoter state *S*_3_ with a probability *q*_3_(*t*). Each promoter state was modelled with ON/OFF switching kinetics of mRNA production of lacZ gene. As suggested by the measurements in [9], the production rates of mRNAs are approximately *ν*_1_ = *ν*_2_ = *ν*_3_ = 15 min^−1^, and the degradation rate is *δ* = 0.5 min^−1^. The constant activation and inactivation rates are

$$\lambda_{1}=0.94\delta ,\;\gamma_{1}=\nu /1.41,\;\lambda_{2}=4.32\delta ,\;\gamma_{2}=\nu /4.08,\;\lambda_{3}=0.81\delta ,\;\gamma_{3}=\nu /2.01,$$

according to the estimations from genetic controls for *P*_*RM*_ activity [22]. The probabilities *q*_1_(*t*), *q*_2_(*t*) and *q*_3_(*t*) were estimated with different levels of lambda repressor concentration [LR]. It was found that *q*_1_ decreased with [LR], while *q*_3_ increased with [LR]. Interestingly, *q*_2_ first increased and then decreased with [LR] and did not surpass 50%.

According to the equilibrium thermodynamic model [21,22,25], we estimate the promoter probabilities *q*_1_(*t*), *q*_2_(*t*) and *q*_3_(*t*) in the following formulae:

4.1 $$q_{1}(t)=\frac{1}{1+a_{1}{\left[\text{LR}(t)\right]}^{2}+a_{2}{\left[\text{LR}(t)\right]}^{4}},$$

4.2 $$q_{2}(t)=\frac{a_{1}{\left[\text{LR}(t)\right]}^{2}}{1+a_{1}{\left[\text{LR}(t)\right]}^{2}+a_{2}{\left[\text{LR}(t)\right]}^{4}}$$

**Figure 4.**
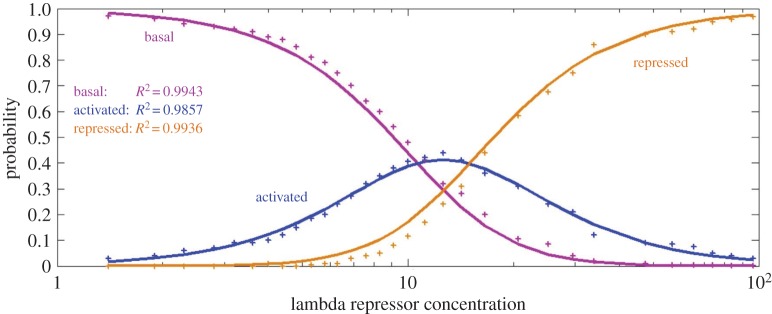
Promoter probabilities versus lambda repressor concentrations. The coloured dots are adapted from experimental data in [22], and the corresponding coloured curves are generated by our estimations (4.1)–(4.3) that provide a rather precise fitting.

4.3 $$\;\text{and}\;q_{3}(t)=\frac{a_{2}{\left[\text{LR}(t)\right]}^{4}}{1+a_{1}{\left[\text{LR}(t)\right]}^{2}+a_{2}{\left[\text{LR}(t)\right]}^{4}},$$

where *a*_1_ = 8.8 × 10^−3^ and *a*_2_ = 3.922 × 10^−5^. As shown in figure 4, these estimations provide a rather precise fitting to the experimental data of [22], with the coefficients of determination (*R*^2^) 0.9943, 0.9857 and 0.9936 for *q*_1_(*t*), *q*_2_(*t*) and *q*_3_(*t*), respectively.

**Figure 5.**
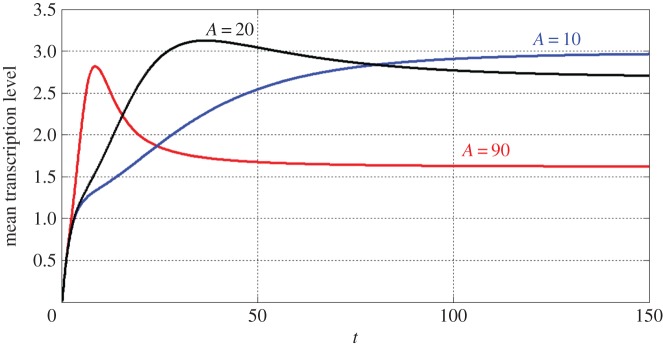
The transcription level *m*(*t*) of lacZ gene responses to monotonically increasing lambda repressor concentration [LR(*t*)]. The transcription level *m*(*t*) either increases monotonically for all time or increases to reach a peak and then goes down.

We investigate how the dynamics of [LR(*t*)] can be carried over to the transcription level *m*(*t*) of lacZ gene. We consider the case where [LR(*t*)] increases in *t* and approaches the steady-state value *A* in response to rapid external stimuli. Since lambda repressor is much more stable than the corresponding mRNA molecules, we follow the work of Thattai *et al.* [33] and assume

$$[\text{LR}(t)]=A(1-e^{-Dt}),$$

where *D* is the degradation rate of lambda repressor. As suggested by [22,34], we estimate that *A* varies within (0, 100] and the degradation rate *D* = 0.033 min^−1^. We will focus on how the steady-state value *A* affects the time-evolution of the transcription level *m*(*t*) of lacZ gene. As figure 5 shows, *m*(*t*) either increases monotonically for all time or increases to reach a peak and then goes down. In the up-down case, for lower *A* (for example, *A* = 20 in figure 5), *m*(*t*) continues to increase and then slowly goes down to the steady-state value m*. We see also that *m*(*t*) increases rapidly and then decreases quickly when lambda repressor concentration reaches a high steady-state value, for example, *A* = 90. Temporal profiles of transcription levels of immune response genes with similar fashions have been observed in recent signal-dependent experiments [3,18].

### 4.2. Transcription dynamics activated by oscillatory signals

Periodic oscillations such as circadian clock and cell cycle are ubiquitous phenomena in life. It has been found that cells can transform certain environmental signals into oscillatory cell signals with specific frequencies and magnitudes to regulate gene expression [4,6,7,19], thereby determining cell function and cell fate. Some transcription factors such as p53 and NF-*κ*B develop oscillatory dynamics in response to distinct stimuli [6,7,23]. For example, it was found that, under periodic Nutlin-3 treatment, the expression level of p53 in MCF-7 breast carcinoma cells followed approximately

4.4 [p53(t)]=1−cos⁡(2πt/3)

that oscillate periodically with a period of 3 h [23].

We are interested in the following important question: How does the transcription level respond when the gene is activated by an oscillatory or periodic signal? A complete answer can be rather complicated because it also depends on the promoter state probabilities and the kinetic rates. Here we use our model to focus on one aspect of the question only: Can multiple pathways filter out signal oscillation to produce a similar transcription dynamics as those activated by constant signals? We first use an example to offer a glimpse on the subtle relation between the temporal variations of the activation signal and the transcription output, including a positive answer to the question. Although the example is not tied to a specific set of experimental data for mathematical clarity, it is motivated by recent experiments in [4,6,7,19,23,35]. The observations from the example will be further testified and reinforced by the transcription of a gene in MCF-7 cells [23].

In the example, we assume that a gene is regulated by two transcription modes sharing the same mRNA production rate *ν* = 15 min^−1^ and degradation rate *δ* = 0.5 min^−1^. The transcription is stimulated by a periodic signal with the intensity quantified by

**Figure 6.**
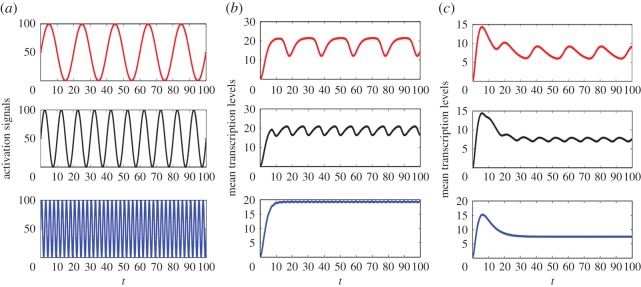
The distinct transcription profiles induced by periodic signals with different frequencies. (*a*) The periodic signals defined by (4.5) with *ω* = 0.1*π*, 0.2*π*, 0.8*π*. (*b*) The mean transcription level *m*(*t*) obtained by solving the system of differential equations in the electronic supplementary material, supplemented with (4.5)–(4.7) and the mRNA production and degradation rates *ν* = 15 min^−1^ and *δ* = 0.5 min^−1^. (*c*) The mean level *m*(*t*) obtained by replacing (4.7) with (4.8) in (*b*).

4.5 [S(t)]=50 sin ωt+50,

a trigonometry function as in (4.4) with an angular frequency *ω* > 0. In figure 6, it is sketched for *ω* = 0.1*π*, 0.2*π*, 0.8*π* and *t* ∈ [0, 100] in red, black and blue, respectively. We specify the promoter probabilities by

4.6 $$q_{1}(t)=\frac{1}{1+0.01{\left[\text{S}(t)\right]}^{2}}\;\text{and}\;q_{2}(t)=\frac{0.01{\left[\text{S}(t)\right]}^{2}}{1+0.01{\left[\text{S}(t)\right]}^{2}},$$

as suggested by the equilibrium thermodynamic model [21,22,25]. Let the activation rates and the inactivation rates of the two promoter states be given by

4.7 $$\lambda_{1}=0.8,\;\gamma_{1}=5,\;\lambda_{2}=1.3\;\text{and}\;\gamma_{2}=0.5,$$

all in the same unit, min^−1^. Then we can obtain the mean transcription level *m*(*t*) by solving the corresponding system of differential equations in the electronic supplementary material, supplemented with (4.5)–(4.7) and the mRNA production and degradation rates *ν* = 15 min^−1^ and *δ* = 0.5 min^−1^. In figure 6*b*, *m*(*t*) is shown for the same values of *ω* in the same range of *t* ∈ [0, 100] as in figure 6*a*. By comparing the signal profile in figure 6*a* with the growth pattern of *m*(*t*) shown in figure 6*b* and many additional examples of *m*(*t*) we examined, including for instance the one shown in figure 6*c* with

4.8 $$\lambda_{1}=0.5,\;\gamma_{1}=0.21,\;\lambda_{2}=0.1\;\text{and}\;\gamma_{2}=0.48,$$

we can make the following observations: (1) *After a short time period of rapid growth, m(t) starts oscillation that turns out to be periodic later with the same period of the signal*. (2) *The oscillation magnitude of m(t) decreases in the signal oscillation frequency *ω**. (3) As shown in the bottom panels of figure 6*b*,*c*, *the oscillation magnitude of m(t) becomes very small and the oscillation is almost completely damped when *ω* ≥ 0.8, for which m(t) behaves as if the transcription is activated by constant signals*.

The two growth curves of *m*(*t*) in the bottom panels of figure 6*b*,*c* resemble the two typical growth patterns generated by the classical two-state model with a single promoter state and constant kinetic rates: *m*(*t*) either increases monotonically for all *t* > 0 as in figure 6*b*, or peaks uniquely and decreases thereafter as in figure 6*c*. The observed resemblance gives a theoretical proof that *the cross-talking of multiple pathways, or more generally, multiple promoter states, can potentially filter out not only the oscillation in activation signals but also the randomness in the selection of promoter states*. We now use the transcription of C12orf5/TIGAR gene in MCF-7 cells as an example to further testify this interesting finding. In its promoter region, there are two p53 binding sites [36]: the low-affinity one upstream of the first exon (BS1) and the high-affinity one within the first intron (BS2). The transcription factor p53 regulates the transcription of C12orf5/TIGAR gene in two promoter states corresponding to p53-BS1 and p53-BS2 binding configurations. According to [23], we estimate the promoter probabilities *q*_1_(*t*) and *q*_2_(*t*) by

4.9 $$q_{1}(t)=\frac{9}{9+{\left[p53(t)\right]}^{2}}\;\text{and}\;q_{2}(t)=\frac{{\left[p53(t)\right]}^{2}}{9+{\left[p53(t)\right]}^{2}};$$

the mRNA production rates at approximately *ν*_1_ = *ν*_2_ = 1 h^−1^; and the decay rates at *δ*_1_ = *δ*_2_ = 0.18 h^−1^. We take the activation and inactivation rates, in the same unit h^−1^, at

**Figure 7.**
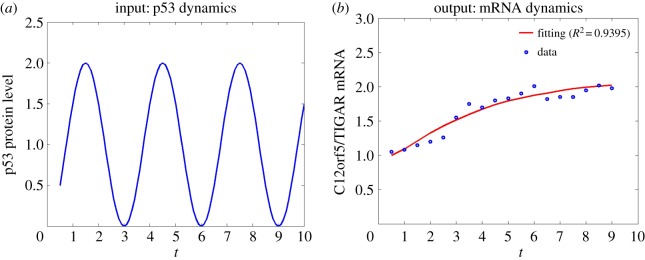
The filtration of the oscillation in periodic signals by multiple promoter states. (*a*) The pulsatile temporal profile of p53 input defined by (4.4). (*b*) The circles are adapted from the experimental data in [23], and the curve is generated by solving equations (2.9)–(2.11) supplemented with (4.9) and (4.10).

4.10 $$\lambda_{1}=0.950,\;\lambda_{2}=0.190,\;\gamma_{1}=0.951\;\text{and}\;\gamma_{2}=2.900,$$

which are within the previously estimated parameter regions for mammalian cells [27,37]. By substituting these data into the corresponding equations in the electronic supplementary material, along with the periodic p53 signal defined in (4.4) (see figure 7*a*), we can determine uniquely the mean transcription level *m*(*t*). As shown in figure 7*b*, the function *m*(*t*) determined by our model increases monotonically and fits extremely well with the measured data from [23] with the coefficient of determination *R*^2^ = 0.9395. It shows that the two p53 binding promoter states filter out the periodic oscillation of p53 and the random switching between the promoter states, and offers an experimental validation of our theoretical prediction. It also offers an alternative explanation of the filtration of periodic p53 signals that was previously attributed to the mRNA decay rates in [23,35].

## 5. Discussion

Cells in living organisms are constantly exposed to environmental or intracellular challenges. Gene transcription regulation is a central process in cells to adapt themselves for these challenges by up- or downregulating the production of mRNA molecules [1,2]. Upon environmental stimuli, the transcription of inducible genes is ultimately achieved through the activation of one or multiple promoter states [21,22]. In this work, we develop a multiple promoter-state model to explore how the nonlinear transcription dynamics is linked to the temporal variations of activation signals.

When the signals inducing the transcription of a gene are stable so that the kinetic rates and the promoter probabilities are all constants, our analysis yields two distinct scenarios for the mean transcription level *m*(*t*): (1) When only the activation rates differ among the promoter states, our theorem 3.1 shows that the presence of multiple promoter states brings on no significant change to the temporal profile of *m*(*t*). It either grows for all *t* > 0 or peaks uniquely as predicted by the classical two-state model with a single promoter state. (2) When the inactivation rates also change with promoter states, *m*(*t*) can develop rather complicated dynamics. Our theorem 3.3 proves that *m*(*t*) may develop a biphasic growth pattern with two peaks even when there are only two promoter states. This indicates that inactivation rates play a more prominent role than activation rates in shaping the temporal profiles of mRNA level in the rewire of transcriptional programmes. Both cellular signals and regulatory factors can actively control the inactivation rates, which may increase in the concentration of transcription repressor [38] or decrease in the concentration of activators [39]. Some transcription factors such as Myc can mediate transcription initiation and elongation bidirectionally, and therefore affect both the activation and inactivation rates [40].

When the gene activation signals change in time, the promoter state probabilities may also change accordingly and a primary step is to determine the probabilities in terms of the signal strength. We followed the approach of the equilibrium thermodynamic model [21,22,25], and our estimation of the probabilities for the transcription of lacZ gene in *Escherichia coli* provides a rather precise fitting, with the average coefficient of determination *R*^2^ > 0.99, to the experimental data of [22]. In general, the dynamic complexity of activation signals can be carried over to the transcription level and *m*(*t*) is expected to develop a complex dynamics as well [4,6,7,19]. We focused on periodic signals that are ubiquitous in gene expression regulation [7,23]. Surprisingly, we found that an orchestrated interplay between multiple promoter states and periodic signals may produce very simple transcription dynamics such as a monotone growth for all *t* > 0. Under the activation of a periodic signal with an oscillation frequency *ω* > 0, our simulation shows that *m*(*t*) grows rapidly in a short time period and then oscillates periodically in the same frequency *ω*. The oscillation magnitude of *m*(*t*) decreases in *ω* and the oscillation is almost completely damped when *ω* becomes large, for which *m*(*t*) behaves as if the transcription is activated by constant signals. The attenuation in the oscillation of *m*(*t*) was further testified by the transcription of C12orf5/TIGAR gene in MCF-7 cells [23]. The function *m*(*t*) determined by our model increases monotonically and fits extremely well with the data from [23] with *R*^2^ = 0.9395. Our interesting finding indicates that multiple signal transduction pathways, or more generally, multiple promoter states, are capable of filtering out both the oscillation in activation signals and the noise in the random switching between the promoter states and producing simple transcription dynamics as in the case of a single promoter state activated by constant signals.

Our analysis shows that the mean transcription level does not always mirror the dynamics of the activation signals. Counterintuitively, it sometimes displays exactly the opposite: In the presence of multiple promoter states, the transcription level may develop a biphasic growth with two peaks under the activation of constant signals or simply increases all the time under the activation of periodic signals. The set of promoter states behaves like a conversion hub that transforms cellular signals passing through the gene regulatory circuit to the production of mRNA molecules. It maps signalling dynamics to a broader spectrum of temporal profiles for transcription output. The map is not expected to be injective in general, as similar dynamical behaviours may be observed from distinct signalling sources. Our knowledge on the architectures of gene regulatory networks has been largely enriched due to the tremendous efforts in past years [1,4,33,37]. Our mathematical approach may help develop a theoretical framework to integrate coherently the genetic circuit with the downstream promoter states to elucidate the linkage between the temporal variations of activation signals and the nonlinear transcription dynamics.

There remain many challenges in advancing or extending our theoretical approach. In our hypothesis (H2), the kinetic rates, including the rates of gene activation, inactivation and mRNA production, are assumed to be constants. The temporal dependence of gene transcription on activation signals is attributed to the time dependence of promoter state selection. The constant assumption on the kinetic rates for given promoter states was also made in our previous studies [28–30,41]. There has been little experimental evidence supporting this assumption besides the data from the seminal study of Sepúlveda *et al.* [22]. More experimental data are needed to validate the hypothesis or determine the cellular conditions under which the hypothesis is valid. Under this assumption, the transcription dynamics of inducible genes is essentially determined by the random selection of promoter states. Future estimations on the promoter state probabilities need to incorporate other physiological conditions, such as DNA binding site accessibility and cell cycle stages, in addition to the TF concentrations in the equilibrium thermodynamic model [21,22,25]. By integrating more components and experimental data, it is expected to advance our modelling approach for a more comprehensive understanding of gene transcription regulation.

## Supplementary Material

Supporting information

Reviewer comments

## Data accessibility

Data available from the Dryad Digital Repository: https://doi.org/10.5061/dryad.vv22bs1 [42].
