# Supplementary material for: Linking dynamical complexities from activation signals to transcription responses
Source: R Soc Open Sci. 2019 Mar 27;6(3):190286. doi: 10.1098/rsos.190286 (PMC6458353; doi:10.1098/rsos.190286)
Supplement: Supporting information [file rsos190286supp1.pdf]

SUPPLEMENTARY MATERIAL
"Linking dynamical complexities from activation signals to transcription responses"

Genghong Lin[1,2], Feng Jiao[1,2], Qiwen Sun[1,2], Moxun Tang[2*]
Jianshe Yu[1] and Zhan Zhou[1]

1. Center for Applied Mathematics, Guangzhou University, Guangzhou, 510006, China

2. Department of Mathematics, Michigan State University, East Lansing, MI 48824, USA

1 The equations

1.1 The master equations

Let $t \geq 0$. We define a random process $X(t)$ to specify the state of the target gene with $X(t) = O_i$ if the gene is OFF, but its transcription is activating by the promoter state S_i, and $X(t) = E_i$ if the gene is ON and the transcription is turned on by S_i. Let $M(t)$ denote the copy number of the mRNA molecules within a single cell. Then the master equations consist of the time evolutions of the probabilities

$$P_{0,i}(m,t) = \text{Prob}\{M(t) = m,\, X(t) = O_i\},\ i = 1, 2, \cdots, n,\, m = 0, 1, 2, \cdots, \tag{1.1}$$

and

$$P_{1,i}(m,t) = \text{Prob}\{M(t) = m,\, X(t) = E_i\},\ i = 1, 2, \cdots, n,\, m = 0, 1, 2, \cdots. \tag{1.2}$$

To determine the time evolution of $P_{1,i}(m,t)$, we let $h > 0$ be an infinitesimal time increment, and assume that the transcription is turned on by S_i with m copies of the mRNA molecules at time $t + h$. Then one of the state transition events in Table 1 occurs during time interval $(t, t+h)$. Adding the four probabilities listed in Table 1 gives

$$\begin{aligned} P_{1,i}(m,t+h) = {} & P_{1,i}(m,t) \cdot (1 - \nu_i h)(1 - \gamma_i h)(1 - m\delta h) + P_{1,i}(m+1,t) \cdot (m+1)\delta h \\ & + P_{1,i}(m-1,t) \cdot \nu_i h + P_{0,i}(m,t) \cdot \lambda_i h. \end{aligned} \tag{1.3}$$

*Author for correspondence: Moxun Tang, e-mail: tangm@msu.edu

	Initial State (t)	Terminal State ($t+h$)	Transition Probability
(a)	(E_i, m)	(E_i, m)	$P_{1,i}(m,t)\cdot(1-\nu_i h)(1-\gamma_i h)(1-m\delta h)$
(b)	$(E_i, m+1)$	(E_i, m)	$P_{1,i}(m+1,t)\cdot(m+1)\delta h$
(c)	$(E_i, m-1)$	(E_i, m)	$P_{1,i}(m-1,t)\cdot\nu_i h$
(d)	(O_i, m)	(E_i, m)	$P_{0,i}(m,t)\cdot\lambda_i h$

Table 1: *The initial states at time t and transition probabilities toward the terminal state (E_i, m) at time $t+h$.* If the transcription is turned on by S_i with m copies of the mRNA molecules at $t+h$, then four initial states at time t, listed in (a), (b), (c), and (d), can reach (E_i, m) with a transition probability of order zero or one of h.

After dividing (1.3) by h and then letting $h \to 0$ we obtain

$$\begin{aligned}\frac{dP_{1,i}(m,t)}{dt} &= \lambda_i P_{0,i}(m,t) - \gamma_i P_{1,i}(m,t) + \nu_i[P_{1,i}(m-1,t) - P_{1,i}(m,t)] \\ &\quad + \delta[(m+1)P_{1,i}(m+1,t) - mP_{1,i}(m,t)],\end{aligned} \tag{1.4}$$

where $P_{1,i}(-1,t) = 0$ by convention. Using a similar argument gives the time evolution of $P_{0,i}(m,t)$ as

$$\frac{dP_{0,i}(m,t)}{dt} = -\lambda_i P_{0,i}(m,t) + q_i(t)\sum_{j=1}^{n}\gamma_j P_{1,j}(m,t) + \delta[(m+1)P_{0,i}(m+1,t) - mP_{0,i}(m,t)]. \tag{1.5}$$

If we assume that the gene is inactive in all cells at a specified initial time $t = 0$, and the residual mRNAs at $t = 0$ are neglected, then we have the initial condition

$$P_{0,i}(0,0) = q_i(0), \quad P_{0,i}(m,0) = 0 \quad \text{for} \quad m > 0, \quad \text{and} \quad P_{1,i}(m,0) = 0 \quad \text{for} \quad m \geq 0. \tag{1.6}$$

1.2 The differential equation of the mean transcription level

To derive the differential equation of the mean transcription level $m(t)$, we introduce

$$P_{0,i}(t) = \sum_{m=0}^{\infty} P_{0,i}(m,t) \quad \text{and} \quad P_{1,i}(t) = \sum_{m=0}^{\infty} P_{1,i}(m,t). \tag{1.7}$$

They measure the frequencies that the transcription is regulated or activated by the promoter state S_i, and relate the mean $m(t)$ by

$$m(t) = \mathbf{E}(M(t)) = \sum_{i=1}^{n}\sum_{k=0}^{\infty} k[P_{0,i}(k,t) + P_{1,i}(k,t)]. \tag{1.8}$$

By differentiating the first equality of (1.7) and substituting (1.4), we obtain

$$\frac{dP_{0,i}(t)}{dt} = -\lambda_i P_{0,i}(t) + q_i(t)\sum_{j=1}^{n}\gamma_j P_{1,j}(t) + \delta\sum_{k=0}^{\infty}[(k+1)P_{0,i}(k+1,t) - kP_{0,i}(k,t)]. \tag{1.9}$$

We notice that $\sum_{k=0}^{\infty}[(k+1)P_{0,i}(k+1,t) - kP_{0,i}(k,t)] = 0$. In terms of (1.6), the initial condition of $P_{0,i}(t)$ is given by $P_{0,i}(0) = q_i(0)$. Therefore, we obtain the differential equation

$$\frac{dP_{0,i}(t)}{dt} = -\lambda_i P_{0,i}(t) + q_i(t)\sum_{j=1}^{n}\gamma_j P_{1,j}(t), \quad P_{0,i}(0) = q_i(0). \tag{1.10}$$

Similarly, we find

$$\frac{dP_{1,i}(t)}{dt} = \lambda_i P_{0,i}(t) - \gamma_i P_{1,i}(t), \quad P_{1,i}(0) = 0. \tag{1.11}$$

By differentiating (1.8) with respect to t, and substituting (1.4) and (1.5), we find

$$\begin{aligned}\frac{dm(t)}{dt} &= \sum_{i=1}^{n}\sum_{k=0}^{\infty} k\nu_i[P_{1,i}(k-1,t) - P_{1,i}(k,t)] \\ &\quad + \delta\sum_{i=1}^{n}\sum_{k=0}^{\infty} k(k+1)[P_{0,i}(k+1,t) + P_{1,i}(k+1,t)] \\ &\quad - \delta\sum_{i=1}^{n}\sum_{k=0}^{\infty} k^2[P_{0,i}(k,t) + P_{1,i}(k,t)].\end{aligned} \tag{1.12}$$

We note that

$$\begin{aligned}\sum_{k=0}^{\infty} k\nu_i[P_{1,i}(k-1,t) - P_{1,i}(k,t)] &= \nu_i\sum_{k=0}^{\infty}(k+1)P_{1,i}(k,t) - \nu_i\sum_{k=0}^{\infty}kP_{1,i}(k,t) \\ &= \nu_i\sum_{k=0}^{\infty}P_{1,i}(k,t) = \nu_i P_{1,i}(t).\end{aligned} \tag{1.13}$$

Similarly, we find that the last two terms of (1.12) equals $-\delta m(t)$. By putting the initial condition (1.6) into (1.8) we get $m(0) = 0$. These calculations lead to

$$\frac{dm(t)}{dt} = -\delta m(t) + \sum_{i=1}^{n}\nu_i P_{1,i}(t), \quad m(0) = 0. \tag{1.14}$$

2 The proof of theorem 3.1

Our discussion is based on the following ordering

$$0 < \lambda_1 < \lambda_2 < \cdots < \lambda_{n-1} < \lambda_n,\ q_i(t) = q_i,\ \gamma_i = \gamma,\ \nu_i = \nu,\ i = 1, 2, \cdots, n. \tag{2.1}$$

The probability that the gene is active at a time t is given by

$$P_E(t) = \sum_{i=1}^{n} P_{1,i}(t), \tag{2.2}$$

where $P_{1,i}(t)$ is defined in (1.7). By taking the sum in (1.11), we see from (1.10) that $P_E(t)$ can be solved from the initial value problem of the homogeneous linear system

$$\begin{cases}\frac{dP_{0,k}(t)}{dt} &= -\lambda_k P_{0,k}(t) + q_k\gamma P_E(t), \\ \frac{dP_E(t)}{dt} &= \sum_{i=1}^{n}\lambda_i P_{0,i}(t) - \gamma P_E(t),\end{cases} \tag{2.3}$$

with the initial conditions $P_E(0) = 0$, $P_{0,k}(0) = q_k$ for $k = 1, 2, \cdots, n$, and the matrix of constant coefficients

$$B = \begin{pmatrix} -\lambda_1 & 0 & \cdots & 0 & q_1\gamma \\ 0 & -\lambda_2 & \cdots & 0 & q_2\gamma \\ \cdots & \cdots & \cdots & \cdots & \cdots \\ 0 & 0 & \cdots & -\lambda_n & q_n\gamma \\ \lambda_1 & \lambda_2 & \cdots & \lambda_n & -\gamma \end{pmatrix}. \tag{2.4}$$

Lemma 1. *Let Condition (2.1) hold. Then the set of eigenvalues of the matrix B contains $\alpha_0 = 0$ and n distinct negative numbers, denoted by $-\alpha_1$, $-\alpha_2$, $\cdots$, $-\alpha_n$, with*

$$\lambda_1 < \alpha_1 < \lambda_2 < \alpha_2 < \cdots < \lambda_{n-1} < \alpha_{n-1} < \lambda_n < \alpha_n. \tag{2.5}$$

If $\alpha_1 \geq \gamma$, then $P_E'(t) > 0$ for all $t > 0$. If $\alpha_1 < \gamma$, then there is a unique $t_E > 0$ such that $P_E(t)$ increases in $(0, t_E)$, peaks at t_E, and decreases in (t_E, ∞).

Proof. Let I denote the $(n+1) \times (n+1)$ identity matrix. The eigenvalues of B are the roots of the characteristic equation

$$f_B(x) = \det(xI - B) = 0.$$

For $1 \leq i \leq n$, we multiply the i-th column of $xI - B$ by $q_i\gamma/(x + \lambda_i)$ and add the product to the last column to make the first n entries vanish. It helps us find

$$\begin{aligned} f_B(x) &= (x + \lambda_1)(x + \lambda_2) \cdots (x + \lambda_n) \left[(x + \gamma) - \sum_{i=1}^{n} \frac{q_i \lambda_i \gamma}{x + \lambda_i} \right] \\ &= (x + \gamma) \prod_{i=1}^{n} (x + \lambda_i) - \gamma h(-x); \quad \text{where } h(x) = \sum_{i=1}^{n} q_i \lambda_i \prod_{j \neq i} (\lambda_j - x). \end{aligned} \tag{2.6}$$

From the first expression of (2.6) we find $f_B(0) = 0$, and from the second expression we find

$$f_B(-\lambda_k) = -\gamma h(\lambda_k) = -\gamma q_k \lambda_k \prod_{j \neq k} (\lambda_j - \lambda_k),$$

which is negative for odd number k, and positive for even number k. If n is odd, then $f_B(-\lambda_n) < 0$ and $f_B \to \infty$ as $x \to -\infty$; if n is even, then $f_B(-\lambda_n) > 0$ and $f_B \to -\infty$ as $x \to -\infty$. Hence f_B vanishes at $x = 0$, exactly once within $(-\lambda_{k+1}, -\lambda_k)$ for $k = 1, 2, \cdots, n-1$, and exactly once within $(-\infty, -\lambda_n)$. Denote the eigenvalues of B by $\alpha_0 = 0$, and $-\alpha_1$, $-\alpha_2$, $\cdots$, $-\alpha_n$ with $\alpha_1 < \alpha_2 < \cdots < \alpha_n$. We see clearly that (2.5) holds.

We may solve the initial value problem (2.3) by using standard approaches in the theory of linear ordinary differential equations [1]. Since $P_E(t)$ can be expressed as a linear combination of $\exp(-\alpha_k t)$, $k = 0, 1, 2, \cdots, n$, as all eigenvalues of B are distinct, we find that the Laplace transform provides the best approach to determine $P_E(t)$ [2, 3]. Let $L_k(x)$ denote the transform of $P_{0,k}(t)$, $k = 1, 2, \cdots, n$, and $L_E(x)$ the transform of $P_E(t)$. Then (2.3) is transformed to the linear system of algebraic equations

$$(xI - B)\,(L_1(x), L_2(x), \cdots, L_n(x), L_E(x))^T = (q_1, q_2, \cdots, q_n, 0)^T,$$

where T stands for the transpose. If we replace the last column of $xI - B$ by $(q_1, q_2, \cdots, q_n, 0)^T$, then the new matrix has a determinant value $h(-x)$, that can be obtained by replacing $-q_i\gamma$ with q_i, and $x+\gamma$ with 0 in (2.6). By applying Cramer's rule, we find $L_E(x) = h(-x)/f_B(x)$. As $f_B(x)$ has a leading term x^{n+1}, we have $f_B(x) = x(x+\alpha_1)\cdots(x+\alpha_n)$ and

$$L_E(x) = \frac{h(-x)}{f_B(x)} = \frac{h(-x)}{x(x+\alpha_1)\cdots(x+\alpha_n)}.$$

On the other hand, if we assume $P_E(t) = \sum_{k=0}^n \overline{\beta}_k \exp(-\alpha_k t)$, then its Laplace transform can be expressed alternatively as $L_E(x) = \sum_{k=0}^n \overline{\beta}_k/(x+\alpha_k)$. Hence

$$\frac{h(-x)}{x(x+\alpha_1)\cdots(x+\alpha_n)} = \sum_{k=0}^n \frac{\overline{\beta}_k}{x+\alpha_k} = \frac{\sum_{k=0}^n \overline{\beta}_k \prod_{j\neq k}(x+\alpha_j)}{x(x+\alpha_1)\cdots(x+\alpha_n)},$$

and therefore

$$h(-x) = \sum_{k=0}^n \overline{\beta}_k \prod_{j\neq k}(x+\alpha_j) \quad \Rightarrow \quad h(\alpha_k) = \overline{\beta}_k \prod_{j\neq k}(\alpha_j - \alpha_k),\ k = 0, 1, \cdots, n. \tag{2.7}$$

From (2.6) we see that $h(0) = \lambda_1\lambda_2\cdots\lambda_n$. For $k \geq 1$, we let $\beta_k = -\alpha_k\overline{\beta}_k$ and finally derive

$$P_E(t) = \frac{\lambda_1\lambda_2\cdots\lambda_n}{\alpha_1\alpha_2\cdots\alpha_n} - \sum_{k=1}^n \frac{\beta_k}{\alpha_k} e^{-\alpha_k t}, \quad \beta_k = \frac{h(\alpha_k)}{\prod_{j\neq k}(\alpha_j - \alpha_k)},\ k = 1, 2, \cdots, n. \tag{2.8}$$

As $f_B(-\alpha_k) = 0$, we see from (2.5) and (2.6) that

$$\beta_k = \frac{(\gamma - \alpha_k)\prod_{i=1}^n(\lambda_i - \alpha_k)}{\gamma\prod_{j\neq k}(\alpha_j - \alpha_k)} > 0 \quad \Leftrightarrow \quad \alpha_k > \gamma. \tag{2.9}$$

If $\alpha_1 \geq \gamma$, then $\beta_1 \geq 0$, $\beta_2, \cdots, \beta_n > 0$, and differentiating (2.8) gives immediately

$$P'_E(t) = \sum_{k=1}^n \beta_k e^{-\alpha_k t} > 0$$

for all $t > 0$. If $\alpha_1 < \gamma$, then $\alpha_n > \gamma$, since otherwise $\alpha_1 < \alpha_2 < \cdots < \alpha_n \leq \gamma$, which would imply $\beta_1, \beta_2, \cdots, \beta_{n-1} < 0$, $\beta_n \leq 0$, and $P'_E(t) < 0$ for all $t > 0$. It contradicts $P_E(0) = 0$ and $P_E(t) > 0$ for $t > 0$. Hence there must be an integer j, $2 \leq j < n$, such that

$$\alpha_{j-1} \leq \gamma < \alpha_j, \quad \beta_1, \beta_2, \cdots, \beta_{j-2} < 0,\ \beta_{j-1} \leq 0,\ \beta_j, \cdots, \beta_n > 0. \tag{2.10}$$

Let $g_E(t) = \exp(\alpha_{j-1}t)P'_E(t) = \sum_{k=1}^n \beta_k \exp[(\alpha_{j-1} - \alpha_k)t]$. Then the second equation and the initial condition of (2.3) yield $g_E(0) = P'_E(0) = \sum_{i=1}^n q_i\lambda_i > 0$. For $t > 0$ sufficiently large, $g_E(t)$ is dominated by the first term $\beta_1 \exp[(\alpha_{j-1} - \alpha_1)t]$ and so $\lim_{t\to\infty} g(t) = -\infty$. From (2.5) and (2.10), we have

$$g'_E(t) = \sum_{k=1}^{j-2}(\alpha_{j-1} - \alpha_k)\beta_k e^{(\alpha_{j-1}-\alpha_k)t} - \sum_{k=j}^n(\alpha_k - \alpha_{j-1})\beta_k e^{(\alpha_{j-1}-\alpha_k)t} < 0.$$

Hence there must be some $t_E > 0$ such that $g_E(t)$ and $P'_E(t)$ remain positive in $[0, t_E)$, vanish at t_E, and become negative for $t > t_E$. The last part of the Lemma is thus verified. $\square$

For the polynomial $h(x)$ defined in (2.6), the ordering of λ_i in (2.1) gives $h(\lambda_i) > 0$ if i is odd, and $h(\lambda_i) < 0$ if i is even, $i = 1, 2, \cdots, n$. It follows that all the $n-1$ roots of $h(x) = 0$ are real, distinct, and lie within (λ_1, λ_n). Denote these roots by $x_1 < x_2 < \cdots < x_{n-1}$. Then

$$\lambda_1 < x_1 < \lambda_2 < x_2 < \cdots < \lambda_{n-1} < x_{n-1} < \lambda_n. \tag{2.11}$$

Now we state and prove theorem 3.1 presented in the main context.

Theorem 1. *Let Condition (2.1) hold. If the zero x_1 of $h(x)$ and the eigenvalue $-\alpha_1$ of B satisfy $x_1 \geq \min\{\delta, \alpha_1\}$, then $m'(t) > 0$ for all $t > 0$. If $x_1 < \min\{\delta, \alpha_1\}$, then there is a finite $t_m > 0$ such that $m(t)$ increases for $t \in (0, t_m)$, peaks at t_m, and decreases for $t > t_m$.*

Proof. As $\nu_i = \nu$, we use the definition of $P_E(t)$ given in (2.2) to change (1.14) to

$$\frac{dm(t)}{dt} = -\delta m(t) + \nu P_E(t), \quad m(0) = 0. \tag{2.12}$$

Since $P_E(0) = 0$, (2.12) gives $m'(0) = 0$. From (2.3) it is seen that $P'_E(0) > 0$. So (2.12) further implies $m''(0) = \nu P'_E(0) > 0$. Consequently, *$m(t)$, $m'(t)$, and $m''(t)$ are all positive for $t > 0$ sufficiently small.* To explore their properties when t is large, it would be helpful to obtain an exact form of $m(t)$. As $P_E(t)$ is expressed explicitly in (2.8), we may find $m(t)$ by solving the simple problem (2.12). However, we noticed that simplifying the coefficient of $\exp(-\delta t)$ in this approach is rather involved. Instead, we place (2.12) into System (2.3) to form a new system of $n+2$ linear equations with the coefficient matrix

$$\overline{B} = \begin{pmatrix} B & 0 \\ \overrightarrow{\nu} & -\delta \end{pmatrix}, \quad \overrightarrow{\nu} = (0, 0, \cdots, 0, \nu) \in R^{n+1}.$$

Let $\overline{I}$ denote the $(n+2) \times (n+2)$ identity matrix. Let $L_m(x)$ denote the Laplace transform of $m(t)$. Then the new system is transformed to

$$(x\overline{I} - \overline{B})\,(L_1(x), L_2(x), \cdots, L_n(x), L_E(x), L_m(x))^T = (q_1, q_2, \cdots, q_n, 0, 0)^T.$$

By applying Cramer's rule to this algebraic system, and evaluating the determinants by the same idea as in the proof of Lemma 1, we find

$$L_m(x) = \frac{\nu h(-x)}{(x+\delta) f_B(x)} = \frac{\nu h(-x)}{x(x+\alpha_1)\cdots(x+\alpha_n)(x+\delta)}.$$

The inverse Laplace transform by partial fraction expansion then gives

$$m(t) = \frac{\nu \lambda_1 \lambda_2 \cdots \lambda_n}{\delta \alpha_1 \alpha_2 \cdots \alpha_n} - \sum_{k=1}^{n} \frac{\nu}{\delta - \alpha_k} \frac{\beta_k}{\alpha_k} e^{-\alpha_k t} - \frac{\nu h(\delta)}{\delta \prod_{j=1}^{n} (\alpha_j - \delta)} e^{-\delta t}. \tag{2.13}$$

When δ equals some α_i, (2.13) is not well-defined literally. However, we can still use (2.13) to analyze $m(t)$ by taking limit $\delta \to \alpha_i$.

To confirm the first part of Theorem 1, we start with the interesting equivalence

$$x_1 \geq \alpha_1 \;\Leftrightarrow\; \alpha_1 \geq \gamma. \tag{2.14}$$

Indeed, from (2.5) and (2.11) we see that both x_1 and α_1 lie in (λ_1, λ_2), within which $h(x) > 0$ for $x \in (\lambda_1, x_1)$, and $h(x) < 0$ for $x \in (x_1, \lambda_2)$. It follows that $x_1 \geq \alpha_1 \Leftrightarrow h(\alpha_1) \geq 0$. From the definition of β_k in (2.8) we have $h(\alpha_1) \geq 0 \Leftrightarrow \beta_1 \geq 0$, and therefore verify (2.14) by using the equivalence in (2.9).

Assume $x_1 \geq \min\{\delta, \alpha_1\}$. If $x_1 \geq \alpha_1$, then (2.14) implies $\alpha_1 \geq \gamma$, and Lemma 1 asserts $P_E'(t) > 0$ for all $t > 0$. Now, if it happens that $m'(t_0) = 0$ at some $t_0 > 0$, then differentiating (2.12) gives $m''(t_0) = \nu P_E'(t_0) > 0$, and so $m(t)$ must assume a strict (local) minimum value at t_0. This contradicts our early observation that $m'(t) > 0$ for small $t > 0$, and proves that $m'(t) > 0$ actually holds for all $t > 0$. In the other case when $x_1 < \alpha_1$, the basic assumption $x_1 \geq \min\{\delta, \alpha_1\}$ and the equivalent relation (2.14) imply $\delta \leq x_1 < \alpha_1 < \gamma$. Because $P_E(0) = 0$, $P_E(t) > 0$ for $t > 0$, and $P_E'(t) = \sum_{k=1}^{n} \beta_k \, e^{-\alpha_k t}$, at least one of $\beta_1, \beta_2, \cdots, \beta_n$ must be positive. Hence (2.9) implies $\gamma < \alpha_n$, and we may continue with the condition

$$\delta \leq x_1 < \alpha_1 < \alpha_2 < \cdots < \alpha_j \leq \gamma < \alpha_{j+1} < \cdots < \alpha_n \tag{2.15}$$

for some $1 \leq j < n$. Thus $h(\delta) > 0$, and by using (2.9) again we find $\beta_1, \cdots, \beta_{j-1} < 0$, $\beta_j \leq 0$, and $\beta_{j+1}, \cdots, \beta_n > 0$. Taking derivative in (2.13) gives

$$m'(t) = -\sum_{k=1}^{n} \frac{\nu \beta_k}{\alpha_k - \delta} \, e^{-\alpha_k t} + \frac{\nu h(\delta)}{(\alpha_1 - \delta) \cdots (\alpha_n - \delta)} \, e^{-\delta t}. \tag{2.16}$$

Since $\beta_k(\alpha_j - \alpha_k) < 0$ for all $k \neq j$, by using (2.15) and (2.16) we find that, for all $t > 0$,

$$\left[e^{\alpha_j t} m'(t)\right]' = -\sum_{k \neq j} \frac{\nu \beta_k (\alpha_j - \alpha_k)}{\alpha_k - \delta} \, e^{(\alpha_j - \alpha_k)t} + \frac{\nu h(\delta)}{\prod_{k \neq j} (\alpha_k - \delta)} \, e^{(\alpha_j - \delta)t} > 0.$$

As $m'(0) = 0$, it shows that $e^{\alpha_j t} m'(t) > 0$, and so $m'(t) > 0$, for all $t > 0$.

To verify the second part of the conclusion in Theorem 1, we assume $x_1 < \min\{\delta, \alpha_1\}$. There are three subcases: (i) $x_1 < \alpha_1 < \delta$; (ii) $x_1 < \delta < \alpha_1$; and (iii) $x_1 < \alpha_1 = \delta$. Let (i) hold first. As (2.5) and (2.11) imply $x_1, \alpha_1 \in (\lambda_1, \lambda_2)$, and $h(x) < 0$ in (x_1, λ_2), we obtain $h(\alpha_1) < 0$. By using (2.8) and (2.16), we have

$$\lim_{t \to \infty} e^{\alpha_1 t} m'(t) = -\frac{\nu \beta_1}{\alpha_1 - \delta} = \frac{\nu h(\alpha_1)}{(\delta - \alpha_1)(\alpha_2 - \alpha_1) \cdots (\alpha_n - \alpha_1)} < 0.$$

Thus $m'(t) < 0$ for all $t > 0$ sufficiently large. If (ii) holds, then $h(\delta) < 0$ and (2.16) implies

$$\lim_{t \to \infty} e^{\delta t} m'(t) = \frac{\nu h(\delta)}{(\alpha_1 - \delta)(\alpha_2 - \delta) \cdots (\alpha_n - \delta)} < 0,$$

so again $m'(t) < 0$ for sufficiently large $t > 0$. In the degenerate case (iii) with $\alpha_1 = \delta$, (2.16) is not directly applicable. We treat (iii) as a limiting case as $\delta \to \alpha_1$ and compute

$$
\begin{aligned}
&\lim_{\delta\to\alpha_1}\left(-\frac{\nu\beta_1}{\alpha_1-\delta}\,e^{-\alpha_1 t}+\frac{\nu h(\delta)}{(\alpha_1-\delta)\cdots(\alpha_n-\delta)}\,e^{-\delta t}\right)\\
&=\frac{-\nu}{(\alpha_2-\alpha_1)\cdots(\alpha_n-\alpha_1)}\lim_{\delta\to\alpha_1}\frac{h(\alpha_1)\,e^{-\alpha_1 t}-h(\delta)\,e^{-\delta t}}{\alpha_1-\delta}\\
&=\frac{\nu\,[h(\alpha_1)t-h'(\alpha_1)]}{(\alpha_2-\alpha_1)\cdots(\alpha_n-\alpha_1)}\,e^{-\alpha_1 t}.
\end{aligned}
$$

For t sufficiently large, this limit is negative as $h(\alpha_1) < 0$, and therefore $m'(t)$, dominated by the two terms with exponentials $\exp(-\alpha_1 t)$ and $\exp(-\delta t)$ in (2.16), is negative as well.

To complete the proof of the second part when $x_1 < \min\{\delta, \alpha_1\}$, we first recall that $m'(t) > 0$ for $t > 0$ sufficiently small, and $m'(t) < 0$ for $t > 0$ sufficiently large. Hence there exists a finite $t_m > 0$ such that $m'(t) > 0$ in $(0, t_m)$, and $m'(t_m) = 0$. Then $m''(t_m) \le 0$, and (2.12) gives $P_E'(t_m) = m''(t_m)/\nu \le 0$. It follows that $t_m \ge t_E$ for t_E defined in Lemma 1, and $P_E'(t) < 0$ for all $t > t_m$. By using (2.12) again, we find $[\exp(\delta t)m'(t)]' = \nu\exp(\delta t)P_E'(t)$. Hence for each $t > t_m$, we have

$$
e^{\delta t}m'(t) = \nu\int_{t_m}^{t} e^{\delta s}P_E'(s)\,ds < 0 \quad\Rightarrow\quad m'(t) < 0.
$$

Our proof is thus completed. □

Corollary 1. *Let Condition (2.1) hold. If $\lambda_1 \ge \min\{\delta, \gamma\}$, then $m'(t) > 0$ for all $t > 0$. If $\lambda_2 \le \min\{\delta, \gamma\}$, then $m(t)$ peaks uniquely at a finite time.*

To see how Corollary 1 follows from Theorem 1, we only need to verify

$$
\lambda_1 \ge \min\{\delta,\gamma\} \;\Rightarrow\; x_1 \ge \min\{\delta,\alpha_1\} \quad\text{and}\quad \lambda_2 \le \min\{\delta,\gamma\} \;\Rightarrow\; x_1 < \min\{\delta,\alpha_1\}.
$$

We note again from (2.5) and (2.11) that $x_1, \alpha_1 \in (\lambda_1, \lambda_2)$. If $\lambda_1 \ge \delta$, then apparently $x_1 > \delta$. If $\lambda_1 \ge \gamma$, then $\alpha_1 > \gamma$, and so (2.14) gives $x_1 > \alpha_1$. Thus $\lambda_1 \ge \min\{\delta, \gamma\}$ implies $x_1 \ge \min\{\delta, \alpha_1\}$. The other relation can be verified similarly.

3 The proof of theorem 3.3

Due to the technical complexity, we focus on the simplest case with two promoter states only with the assumption

$$
n = 2,\; q_1(t) = q_1,\; q_2(t) = q_2,\; \gamma_1 \ne \gamma_2,\; \nu_1 = \nu_2 = \nu. \tag{3.1}
$$

In this case, we find that multiple promoter states could generate complex transcription dynamics. Even with two promote states, $m(t)$ may develop a biphasic growth pattern with two peaks not seen in the classical two-state model.

We rewrite (1.10) and (1.11) as

$$\begin{cases} \frac{dP_{0,1}(t)}{dt} = -\lambda_1 P_{0,1}(t) + q_1\gamma_1 P_{1,1}(t) + q_1\gamma_2 P_{1,2}(t) \\ \frac{dP_{0,2}(t)}{dt} = -\lambda_2 P_{0,2}(t) + q_2\gamma_1 P_{1,1}(t) + q_2\gamma_2 P_{1,2}(t) \\ \frac{dP_{1,1}(t)}{dt} = \lambda_1 P_{0,1}(t) - \gamma_1 P_{1,1}(t) \\ \frac{dP_{1,2}(t)}{dt} = \lambda_2 P_{0,2}(t) - \gamma_2 P_{1,2}(t) \end{cases} \tag{3.2}$$

with the initial conditions $P_{0,1}(0) = q_1$, $P_{0,2}(0) = q_2$ and $P_{1,1}(0) = P_{1,2}(0) = 0$, and the matrix of constant coefficients

$$C = \begin{pmatrix} -\lambda_1 & 0 & q_1\gamma_1 & q_1\gamma_2 \\ 0 & -\lambda_2 & q_2\gamma_1 & q_2\gamma_2 \\ \lambda_1 & 0 & -\gamma_1 & 0 \\ 0 & \lambda_2 & 0 & -\gamma_2 \end{pmatrix}. \tag{3.3}$$

It can be verified that zero is a simple eigenvalue of C. Let $-\alpha_1$, $-\alpha_2$, and $-\alpha_3$ denote the three nonzero eigenvalues of C, which are the roots of the cubic equation

$$x^3 + b_1x^2 + b_2x + b_3 = 0$$

with

$$b_1 = \lambda_1 + \lambda_2 + \gamma_1 + \gamma_2, \quad b_2 = \lambda_1\lambda_2 + \lambda_1\gamma_2 + \lambda_2\gamma_1 + \gamma_1\gamma_2 + q_1\lambda_2\gamma_2 + q_2\lambda_1\gamma_1,$$

and

$$b_3 = q_1\lambda_2\gamma_1\gamma_2 + q_1\lambda_1\lambda_2\gamma_2 + q_2\lambda_1\lambda_2\gamma_1 + q_2\lambda_1\gamma_1\gamma_2.$$

Let

$$c_1 = -\frac{b_1^3}{27} + \frac{b_1b_2}{6} - \frac{b_3}{2} \quad \text{and} \quad c_2 = \left(\frac{b_1^2}{9} - \frac{b_2}{3}\right)^3.$$

We note that $c_1c_2 \neq 0$. If $c_1^2 < c_2$, then there exists $\theta_0 \in (0, \pi/3)$ such that $\cos(3\theta_0) = c_1/\sqrt{c_2}$. In this case, α_1, α_2, and α_3 can be respectively written as

$$\alpha_1 = \frac{b_1}{3} - 2\sqrt[6]{c_2}\cos\theta_0, \quad \alpha_2 = \frac{b_1}{3} - 2\sqrt[6]{c_2}\cos(\theta_0 + 4\pi/3),$$

and

$$\alpha_3 = \frac{b_1}{3} - 2\sqrt[6]{c_2}\cos(\theta_0 + 2\pi/3).$$

It is not hard to show that they are distinct positive real numbers with

$$\alpha_1 < \alpha_2 < \alpha_3. \tag{3.4}$$

If $c_1^2 = c_2$, then $c_1 = \pm\sqrt{c_2}$. By taking the forms above, we have that either $\alpha_1 = b_1/3 - 2\sqrt[6]{c_2}$ and $\alpha_2 = \alpha_3 = b_1/3 + \sqrt[6]{c_2}$ when $c_1 = \sqrt{c_2}$, or $\alpha_3 = b_1/3 - 2\sqrt[6]{c_2}$ and $\alpha_1 = \alpha_2 = b_1/3 + \sqrt[6]{c_2}$ when $c_1 = -\sqrt{c_2}$. Thus, by labelling α_1, α_2, and α_3 appropriately, we have $0 < \alpha_1 < \alpha_2 = \alpha_3$.

If $c_1^2 > c_2$, then two of α_1, α_2, and α_3 become conjugate complex numbers and the remaining one is positive. Let x_1 and x_2 denote the two roots of

$$H(x) = \nu q_1 \lambda_1 (\lambda_2 - x)(\gamma_2 - x) + \nu q_2 \lambda_2 (\lambda_1 - x)(\gamma_1 - x) = 0. \tag{3.5}$$

It is possible that both x_1 and x_2 are complex numbers.

Now we state and prove theorem 3.3 given in the main context.

Theorem 2. *Let Conditions (3.1) and (3.4) hold. Assume that $\delta \neq \alpha_i$ for $i = 1, 2, 3$. If x_1 and x_2 are complex valued, then either $m(t)$ increases monotonically for all $t > 0$, or $m(t)$ develops two peaks in a biphasic growth. If x_1 and x_2 are real valued with $x_1 < x_2 < \alpha_3$, then we have:*

(1) *If either $x_1 < \alpha_1 < x_2$ and $x_1 < \delta$, or $x_1 < \delta < x_2 < \alpha_1$, then $m(t)$ increases initially until reaching a peak and then goes down.*

(2) *If one of the following occurs:* (i) $\delta < x_1 < \alpha_1 < x_2$, (ii) $\alpha_1 < x_1 < \delta < x_2 < \alpha_2$, (iii) $\alpha_2 < \delta < x_1$, *and* (iv) $\alpha_1 < x_1 < \alpha_2 < x_2$, *then $m'(t) > 0$ for all $t > 0$.*

(3) *If one of the following occurs:* (i) $x_2 < \min\{\alpha_1, \delta\}$, (ii) $\max\{\alpha_2, \delta\} < x_1$, (iii) $\max\{\alpha_1, \delta\} < x_1 < x_2 < \alpha_2$, (iv) $\delta < x_1 < x_2 < \alpha_1$, (v) $\alpha_2 < x_1 < x_2 < \delta$, (vi) $\alpha_1 < x_1 < x_2 < \min\{\alpha_2, \delta\}$, *then either $m(t)$ increases monotonically for all $t > 0$, or $m(t)$ develops two peaks in a biphasic growth.*

Proof. As $n = 2$, we rewrite (1.14) as

$$\frac{dm(t)}{dt} = -\delta m(t) + \nu P_{1,1}(t) + \nu P_{1,2}(t), \quad m(0) = 0. \tag{3.6}$$

Since $P_{1,1}(0) = P_{1,2}(0) = 0$, (3.6) gives $m'(0) = 0$. From (3.2) it is seen that $P'_{1,1}(0) > 0$ and $P'_{1,2}(0) > 0$. Hence (3.6) further implies $m''(0) = \nu P'_{1,1}(0) + \nu P'_{1,2}(0) > 0$. Consequently, *$m(t)$, $m'(t)$, and $m''(t)$ are all positive for $t > 0$ sufficiently small.* In what follows, we first derive the exact form of $m(t)$ by using the Laplace transform. We place (3.6) into System (3.2) to form a new system of linear equations with the coefficient matrix

$$\overline{C} = \begin{pmatrix} C & 0 \\ V & -\delta \end{pmatrix}, \quad V = (0, 0, \nu, \nu).$$

Let $\overline{I}$ denote the 5×5 identity matrix. Let $L_{0,1}(x)$, $L_{0,2}(x)$, $L_{1,1}(x)$, $L_{1,2}(x)$, and $L_m(x)$ denote respectively the Laplace transforms of $P_{0,1}(t)$, $P_{0,2}(t)$, $P_{1,1}(t)$, $P_{1,2}(t)$, and $m(t)$. Then the new system is transformed to

$$(x\overline{I} - \overline{C}) \left(L_{0,1}(x), L_{0,2}(x), L_{1,1}(x), L_{1,2}(x), L_m(x)\right)^T = (q_1, q_2, 0, 0, 0)^T.$$

By applying Cramer's rule to this algebraic system, we find

$$L_m(x) = \frac{H(-x)}{x(x+\alpha_1)(x+\alpha_2)(x+\alpha_3)(x+\delta)}.$$

Similar to the derivation of (2.8), the inverse Laplace transform by partial fraction expansion then gives

$$m(t) = \frac{\nu}{\delta} \cdot \frac{q_1\lambda_1\lambda_2\gamma_2 + q_2\lambda_1\lambda_2\gamma_1}{\alpha_1\alpha_2\alpha_3} - \frac{\beta_\delta}{\delta}e^{-\delta t} - \frac{\beta_1}{\alpha_1}e^{-\alpha_1 t} - \frac{\beta_2}{\alpha_2}e^{-\alpha_2 t} - \frac{\beta_3}{\alpha_3}e^{-\alpha_3 t}, \tag{3.7}$$

where

$$\begin{aligned} \beta_\delta &= \frac{H(\delta)}{(\alpha_1-\delta)(\alpha_2-\delta)(\alpha_3-\delta)}, \ \beta_1 = \frac{H(\alpha_1)}{(\delta-\alpha_1)(\alpha_2-\alpha_1)(\alpha_3-\alpha_1)}, \\ \beta_2 &= \frac{H(\alpha_2)}{(\delta-\alpha_2)(\alpha_1-\alpha_2)(\alpha_3-\alpha_2)}, \ \beta_3 = \frac{H(\alpha_3)}{(\delta-\alpha_3)(\alpha_1-\alpha_3)(\alpha_2-\alpha_3)}. \end{aligned} \tag{3.8}$$

Thus

$$m'(t) = \beta_\delta e^{-\delta t} + \beta_1 e^{-\alpha_1 t} + \beta_2 e^{-\alpha_2 t} + \beta_3 e^{-\alpha_3 t}. \tag{3.9}$$

In the following, we present the proof for the case $\delta < \alpha_1$, since the other case $\alpha_1 < \delta$ can be treated similarly. If x_1 and x_2 are complex valued, then $H(x) > 0$ for all x. In view of $\delta < \alpha_1 < \alpha_2 < \alpha_3$, we obtain

$$\beta_\delta > 0, \ \beta_1 < 0, \ \beta_2 > 0, \ \beta_3 < 0. \tag{3.10}$$

For $t > 0$ sufficiently large, $m'(t)$ is dominated by $\beta_\delta \exp(-\delta t)$ in (3.9) and is therefore positive. It follows from (3.9) that

$$\left[e^{\alpha_3 t}m'(t)\right]' = e^{(\alpha_3-\delta)t}g(t), \ g(t) = (\alpha_3-\delta)\beta_\delta + (\alpha_3-\alpha_1)\beta_1 e^{(\delta-\alpha_1)t} + (\alpha_3-\alpha_2)\beta_2 e^{(\delta-\alpha_2)t}. \tag{3.11}$$

It is seen that

$$g(0) = m''(0) > 0 \quad \text{and} \quad \lim_{t\to\infty} g(t) = (\alpha_3-\delta)\beta_\delta > 0. \tag{3.12}$$

We can see from (3.11) that

$$m'(t) = e^{-\alpha_3 t}G(t), \text{ where } G(t) = \int_0^t e^{(\alpha_3-\delta)s}g(s)ds, \ t > 0. \tag{3.13}$$

It follows from (3.9) and (3.13) that

$$G(0) = 0, \quad \lim_{t\to\infty} G(t) = +\infty, \quad \text{and} \quad G'(t) = e^{(\alpha_3-\delta)t}g(t). \tag{3.14}$$

Differentiating $g(t)$ gives

$$g'(t) = \frac{H(\alpha_1)}{\alpha_2-\alpha_1}e^{(\delta-\alpha_2)t}\left[e^{(\alpha_2-\alpha_1)t} - \frac{H(\alpha_2)}{H(\alpha_1)}\right].$$

If $H(\alpha_2) \le H(\alpha_1)$, then $g'(t) \ge 0$, and so $g(t) > 0$ for all $t > 0$. Hence (3.13) gives $m'(t) > 0$ for all $t > 0$. If $H(\alpha_2) > H(\alpha_1)$, then $g'(t) < 0$ in $(0, t_0)$ for

$$t_0 = \frac{1}{\alpha_2-\alpha_1}\left[\ln\left(H(\alpha_2)\right) - \ln\left(H(\alpha_1)\right)\right],$$

and becomes positive for $t > t_0$. If $g(t_0) \geq 0$, then $g(t) > 0$ for all $t > 0$ and $t \neq t_0$. Thus (3.13) gives again $m'(t) > 0$ for all $t > 0$. If $g(t_0) < 0$, then (3.12) implies that $g(t)$ has two zeros $t_1 > 0$ and $t_2 > 0$ with $g(t) > 0$ in $(0, t_1) \cup (t_2, +\infty)$, and $g(t) < 0$ in (t_1, t_2). It follows from (3.14) that

$$G'(t) > 0 \text{ for } t \in (0, t_1) \cup (t_2, +\infty), \quad \text{and} \quad G'(t) < 0 \text{ for } t \in (t_1, t_2). \tag{3.15}$$

If $G(t_2) \geq 0$, then $G(t) > 0$ for all $t > 0$ and $t \neq t_2$, and so $m'(t) > 0$ for all $t > 0$ and $t \neq t_2$. We recall that $G(t) > 0$ for $t > 0$ both sufficiently small and large. Thus, if $G(t_2) < 0$, then (3.15) implies that $G(t)$ has two zeros $T_1 > 0$ and $T_2 > 0$ with $G(t) > 0$ in $(0, T_1) \cup (T_2, +\infty)$, and $G(t) < 0$ in (T_1, T_2). It follows from (3.13) that $m'(t) > 0$ in $(0, T_1) \cup (T_2, +\infty)$ and $m'(t) < 0$ in (T_1, T_2). This finishes the proof of the case when x_1 and x_2 are complex valued.

In the following, we assume that x_1 and x_2 are real valued with $x_1 < x_2 < \alpha_3$ and prove the remaining conclusions.

(1) We present the proof with the additional assumption that $x_1 < \delta < \alpha_1 < \alpha_2 < x_2 < \alpha_3$, as the other cases can be dealt with by the same idea. Since

$$x_1 < \delta < \alpha_1 < \alpha_2 < x_2 < \alpha_3 \ \Rightarrow \ \beta_\delta < 0, \ \beta_1 > 0, \ \beta_2 < 0, \ \beta_3 < 0, \tag{3.16}$$

by using (3.9) again, we obtain

$$\left[e^{\alpha_1 t} m'(t)\right]' = e^{(\alpha_1 - \delta)t} f(t), \ f(t) = (\alpha_1 - \delta)\beta_\delta + (\alpha_1 - \alpha_2)\beta_2 e^{(\delta - \alpha_2)t} + (\alpha_1 - \alpha_3)\beta_3 e^{(\delta - \alpha_3)t}, \tag{3.17}$$

with $f(0) = m''(0) > 0$ and $\lim_{t\to\infty} f(t) = (\alpha_1 - \delta)\beta_\delta < 0$. In terms of (3.16) and (3.17), we have $(\delta - \alpha_2)(\alpha_1 - \alpha_2)\beta_2 < 0$ and $(\delta - \alpha_3)(\alpha_1 - \alpha_3)\beta_3 < 0$, and so

$$f'(t) = (\delta - \alpha_2)(\alpha_1 - \alpha_2)\beta_2 e^{(\delta - \alpha_2)t} + (\delta - \alpha_3)(\alpha_1 - \alpha_3)\beta_3 e^{(\delta - \alpha_3)t} < 0.$$

Hence, there exists a $t_3 > 0$ such that $f(t) > 0$ in $(0, t_3)$, $f(t_3) = 0$, and $f(t) < 0$ in $(t_3, +\infty)$. For $t > 0$ sufficiently large, $m'(t)$, dominated by the term with $\exp(-\delta t)$ in (3.9), is negative. We also recall that $m'(t) > 0$ for $t > 0$ sufficiently small. Thus, there exists a finite $t_4 > 0$ such that $m'(t) > 0$ in $(0, t_4)$, and $m'(t_4) = 0$. Then $m''(t_4) \leq 0$, and (3.17) gives $f(t_4) = \exp(\delta t_4) m''(t_4) \leq 0$. It follows that $t_4 \geq t_3$ and $f(t) < 0$ for all $t > t_4$. Hence, by using (3.17) again, for each $t > t_4$, we have

$$m'(t) = e^{-\alpha_1 t} \int_{t_4}^{t} e^{(\alpha_1 - \delta)s} f(s) ds < 0.$$

(2) We present the proof for the case $\delta < x_1 < \alpha_1 < \alpha_2 < x_2 < \alpha_3$, as the other cases can be handled similarly. With this specification, we have

$$\beta_\delta > 0, \ \beta_1 > 0, \ \beta_2 < 0, \ \beta_3 < 0. \tag{3.18}$$

By (3.9) and (3.18), we obtain for all $t > 0$

$$\left[e^{\alpha_2 t} m'(t)\right]' = (\alpha_2 - \delta)\beta_\delta e^{(\alpha_2 - \delta)t} + (\alpha_2 - \alpha_1)\beta_1 e^{(\alpha_2 - \alpha_1)t} + (\alpha_2 - \alpha_3)\beta_3 e^{(\alpha_2 - \alpha_3)t} > 0.$$

As $m'(0) = 0$, it shows that $e^{\alpha_2 t} m'(t) > 0$, and so $m'(t) > 0$ for all $t > 0$.
(3) We give a short description for the proof with the additional condition that $x_1 < x_2 < \delta < \alpha_1 < \alpha_2 < \alpha_3$, as the other cases can be proceeded in an analogous manner. Under this extra condition, (3.10) holds again. Thus the remaining discussion is the same as the proof given above for two complex valued x_1 and x_2, and we omit it here. □

References

[1] Hirsch, M. W., Smale, S., Devaney, R. (2003) Differential equations, dynamical systems, and an introduction to chaos (2nd edn.). San Diego: Academic Press.

[2] Tang, M. (2008) The mean and noise of stochastic gene transcription. J. Theor. Biol., 253(2), 271-280.

[3] Tang, M. (2010) The mean frequency of transcriptional bursting and its variation in single cells. J. Math. Biol., 60(1), 27-58.
