# Supplementary material for: Linking dynamical complexities from activation signals to transcription responses
Source: R Soc Open Sci. 2019 Mar 27;6(3):190286. doi: 10.1098/rsos.190286 (PMC6458353; doi:10.1098/rsos.190286)
Supplement: Reviewer comments [file rsos190286_review_history.pdf]

THE ROYAL SOCIETY
PUBLISHING

ROYAL SOCIETY
OPEN SCIENCE

Linking dynamical complexities from activation signals to transcription responses

Genghong Lin, Feng Jiao, Qiwen Sun, Moxun Tang, Jianshe Yu and Zhan Zhou

Article citation details
R. Soc. open sci. **6**: 190286.
http://dx.doi.org/10.1098/rsos.190286

Review timeline
Original submission: 16 February 2019
Final acceptance: 27 February 2019

Note: Reports are unedited and appear as submitted by the referee. The review history appears in chronological order.

Note: This manuscript was transferred from another Royal Society journal with peer review.

Review History

Decision letter (RSOS-190286)

27-Feb-2019

Dear Dr Tang:

It is a pleasure to accept your manuscript entitled "Linking dynamical complexities from activation signals to transcription responses" in its current form for publication in Royal Society Open Science. The editors have assessed your manuscript and found that the comments of the previous reviewers have been carefully addressed.

Thank you for your fine contribution. On behalf of the Editors of Royal Society Open Science, we look forward to your continued contributions to the Journal.

Kind regards,
Royal Society Open Science Editorial Office
Royal Society Open Science
openscience@royalsociety.org

Reports © 2019 The Reviewers; Decision Letters © 2019 The Reviewers and Editors; Responses © 2019 The Reviewers, Editors and Authors. Published by the Royal Society under the terms of the Creative Commons Attribution License http://creativecommons.org/licenses/by/4.0/, which permits unrestricted use, provided the original author and source are credited

on behalf of Professor Takashi Suzuki (Associate Editor) and Professor Mark Chaplain (Subject Editor).

Follow Royal Society Publishing on Twitter: @RSocPublishing
Follow Royal Society Publishing on Facebook:
https://www.facebook.com/RoyalSocietyPublishing.FanPage/
Read Royal Society Publishing's blog: https://blogs.royalsociety.org/publishing/
